# Solitary Fibrous Tumor of the Nasal Vestibule: A Case Report

**DOI:** 10.7759/cureus.99763

**Published:** 2025-12-21

**Authors:** Akira Nakazono

**Affiliations:** 1 Department of Otolaryngology - Head and Neck Surgery, Faculty of Medicine and Graduate School of Medicine, Hokkaido University, Sapporo, JPN

**Keywords:** endoscopic approach, nab2-stat6 fusion, nasal vestibule, sinonasal tumor, solitary fibrous tumor

## Abstract

Solitary fibrous tumor (SFT) is a rare mesenchymal tumor defined by the NGFI-A binding protein 2 (NAB2)-signal transducer and activator of transcription 6 (STAT6) fusion gene and rarely arises in the sinonasal tract. An origin in the nasal vestibule is exceptionally rare; to our knowledge, there is only one previously documented case in the English literature.

We report a 48-year-old man who presented with progressive right-sided nasal obstruction and external nasal deformity caused by a well-circumscribed, enhancing tumor occupying the nasal vestibule and extending to the inferior turbinate. Computed tomography (CT) and magnetic resonance imaging (MRI) demonstrated a homogeneous, vascular-rich mass without bone erosion, and positron emission tomography-computed tomography (PET-CT) revealed mild fluorodeoxyglucose (FDG) uptake. The lesion was removed endoscopically via piecemeal resection due to the limited vestibular space. Histopathological examination showed a patternless spindle cell proliferation with collagen bundles and branching, staghorn vessels. Immunohistochemistry demonstrated diffuse nuclear STAT6 and CD34 positivity, confirming SFT, with a low Ki-67 labeling index of 5%, and no necrosis or significant mitotic activity. The patient recovered without postoperative complications and continued to be disease-free at the 12-month follow-up visit.

To our knowledge, this case represents the second known example of an SFT originating in the nasal vestibule and highlights that even in anatomically constrained regions where en bloc excision is difficult, meticulous piecemeal endoscopic resection can achieve complete tumor clearance and favorable early outcomes. Long-term surveillance remains essential due to the potential for late recurrence.

## Introduction

A solitary fibrous tumor (SFT) is a rare mesenchymal tumor first reported in the pleura by Klemperer and Coleman in 1931 [1]. It was long regarded as a neoplasm confined to the pleura; however, it is now recognized to occur in virtually any anatomical region, including the peritoneum, meninges, orbit, and sinonasal tract [2,3]. Pleural cases are malignant in 13%-23%, whereas extrapleural cases are mostly benign [4].

In the 2020 WHO Classification of Soft Tissue and Bone Tumors [5], SFT is categorized as an intermediate fibroblastic neoplasm with low metastatic potential, defined by the NGFI-A binding protein 2 (NAB2)-signal transducer and activator of transcription 6 (STAT6) gene fusion [6,7]. Head and neck SFTs account for approximately 5%-27% of all SFTs, with sinonasal involvement being particularly rare [8].

To date, fewer than 50 sinonasal SFTs have been reported in the English literature. Among these, only one previously documented case has arisen from the nasal vestibule, as reported by Fujii et al. in 2015 [9]. Due to its extreme rarity, diagnosis is often challenging and may be confounded by histologic overlap with other spindle cell tumors, such as glomangiopericytoma, schwannoma, and fibrosarcoma. Immunohistochemical demonstration of nuclear STAT6 positivity, reflecting NAB2-STAT6 fusion, is considered a diagnostic hallmark [6,7].

In this report, we present what appears to be the second known case of a nasal vestibular SFT extending to the inferior turbinate and successfully treated with piecemeal endoscopic resection. We also reviewed the relevant literature on sinonasal SFT, with an emphasis on recurrence, metastasis risk, and the exceptional rarity of the nasal vestibular origin.

## Case presentation

**Figure 1:**
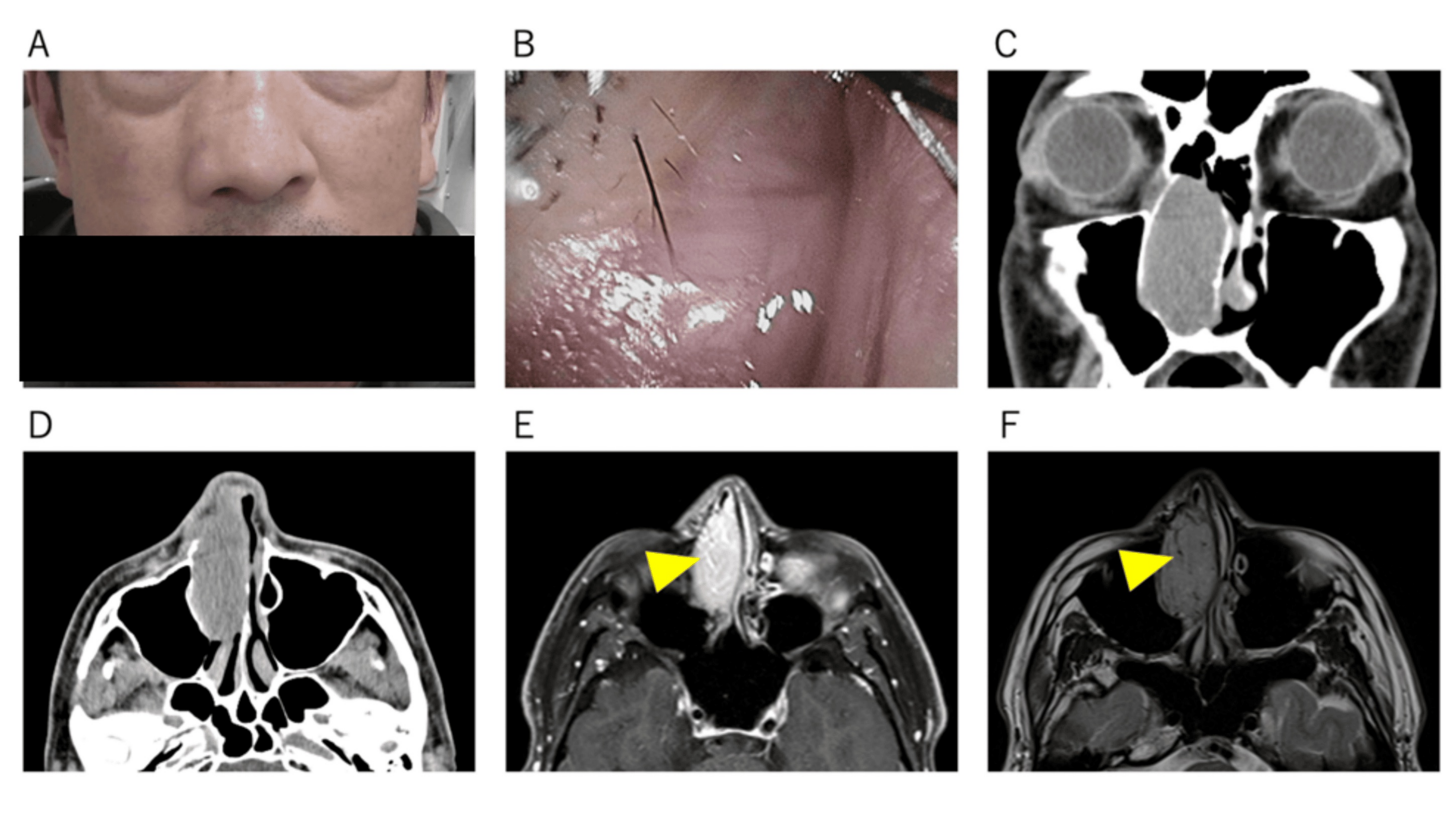
Initial Clinical Findings at Presentation (A) Slight swelling of the right nasal ala and the right side of the nasal root is observed. (B) The right anterior nasal cavity is completely filled by a smooth-surfaced mass. (C-D) CT images show that the tumor occupies the nasal cavity posteriorly without evidence of bony destruction. (E) Gadolinium-enhanced T1-weighted MRI demonstrates marked enhancement with internal tubular structures (yellow arrowhead). (F) On T2-weighted imaging, the tumor appears isointense with internal flow voids (yellow arrowhead). CT, computed tomography; MRI, magnetic resonance imaging

A 48-year-old man presented with progressive right-sided nasal obstruction and an external nasal deformity that had developed gradually over several years. The patient denied epistaxis, facial pain, or olfactory disturbance. He had no history of trauma, nasal surgery, or occupational exposure to dust or carcinogenic agents. The patient’s family history was unremarkable. At the initial visit, the right nasal ala and the right side of the nasal root were slightly swollen due to the tumor (Figure 1A). On anterior rhinoscopy and nasal endoscopy, a firm, smooth, non-tender mass completely occluded the right anterior nasal cavity (Figure 1B). The overlying mucosa appeared normal, and imaging studies revealed a well-defined lesion confined to the right nasal cavity.

Computed tomography (CT) images revealed a homogeneous soft-tissue mass compressing the adjacent structures and displacing the nasal septum toward the contralateral side, without bony erosion or infiltration (Figures 1C, 1D). Magnetic resonance imaging (MRI) revealed a mildly lobulated, well-demarcated mass. Compared with the nasal mucosa, the lesion exhibited iso- to slightly hyperintense signals on T1-weighted images (T1WIs) and heterogeneous hypointensity on T2-weighted images (T2WIs). Diffusion-weighted imaging (DWI) revealed an isointense signal, whereas gadolinium-enhanced T1WI revealed homogeneous enhancement. Internal linear flow voids and strongly enhanced vascular structures were noted, consistent with feeding vessels (Figures 1E, 1F).

Positron emission tomography-computed tomography (PET-CT) revealed mild fluorodeoxyglucose (FDG) uptake with an SUVmax of 2.2 and no interval increase between the early and late phases. No cervical lymphadenopathy or distant metastasis was detected, and the lesion was endoscopically resected under general anesthesia. Because of the limited nasal vestibular space and tumor size, piecemeal resection was performed following internal debulking, allowing for complete exposure and dissection under direct endoscopic visualization. The anterior segment of the inferior turbinate was resected together with the tumor, with careful attention to avoid injury to the nasolacrimal duct orifice. The tumor was completely removed macroscopically, and intraoperative bleeding was minimal. The patient experienced a smooth recovery after surgery and was discharged on day 6.

**Figure 2:**
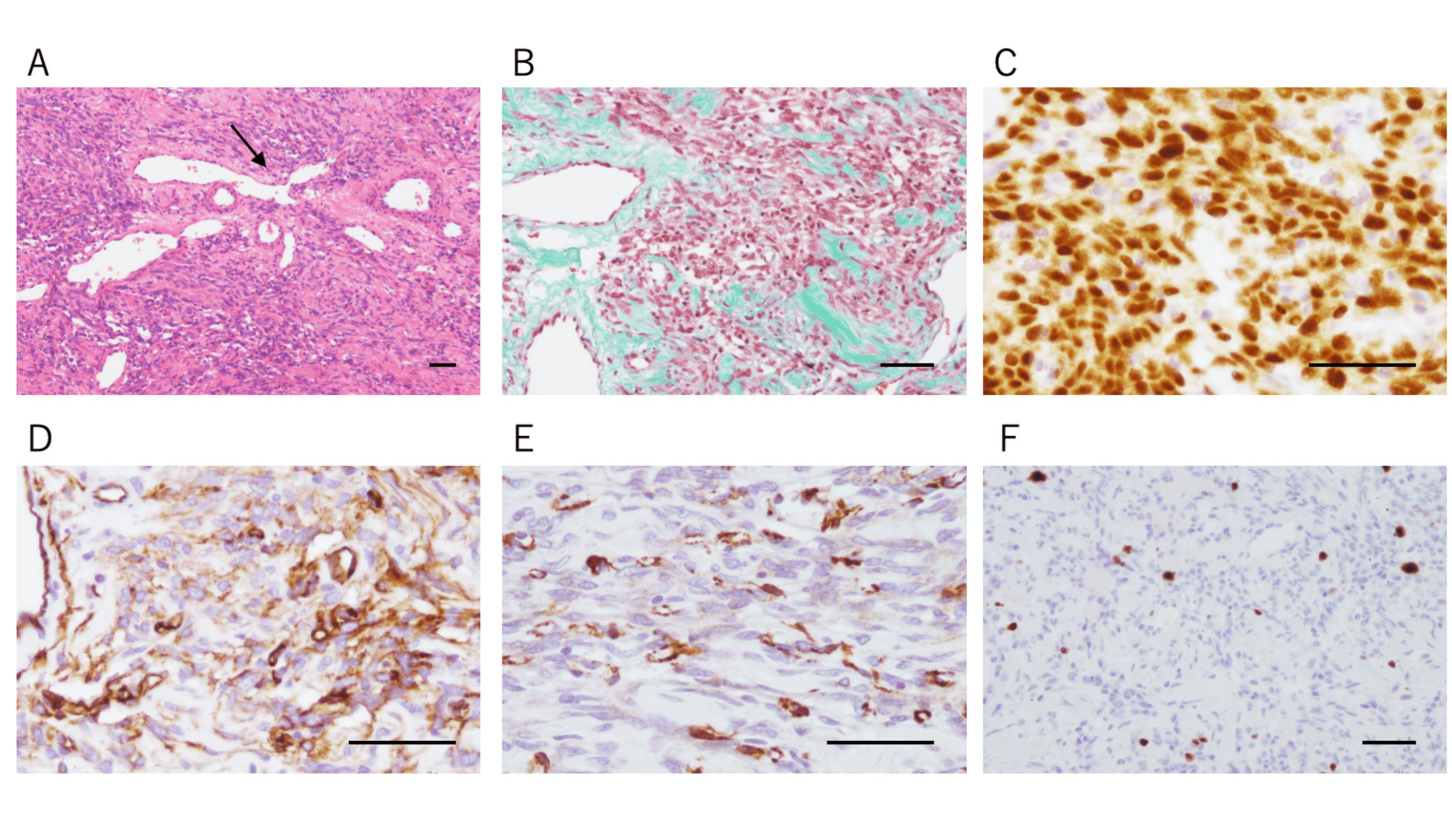
Histopathologic Features of the Tumor (A) Hematoxylin-eosin staining showing proliferation of short spindle cells arranged in a patternless architecture, with branching (“staghorn-like”) vessels (black arrow). (B) Elastica-Masson staining demonstrates increased collagen deposition within the stroma. (C) Nuclear signal transducer and activator of transcription 6 (STAT6) expression is positive. (D) CD34 is positive. (E) α-smooth muscle actin (α-SMA) is focally and weakly positive. (F) Ki-67 labeling index is approximately 5%. All scale bars represent 50 µm.

Histopathological examination revealed spindle-shaped cells arranged in a “patternless” architecture, with alternating hypercellular and hypocellular areas interspersed with collagen bundles and branching (“staghorn”) vessels (Figure 2A). Elastica-Masson staining further demonstrated prominent collagen fibers within the tumor stroma, corroborating the presence of abundant collagen bundles noted on routine histology (Figure 2B). No necrosis or significant mitotic activity was observed. Immunohistochemistry revealed diffuse positivity for nuclear STAT6 (Figure 2C) and CD34 (Figure 2D), confirming SFT. α-smooth muscle actin (α-SMA) was focally and weakly positive (Figure 2E), whereas CD31, desmin, and S-100 were negative. The Ki-67 labeling index was approximately 5%, indicating low proliferative activity (Figure 2F).

**Figure 3:**
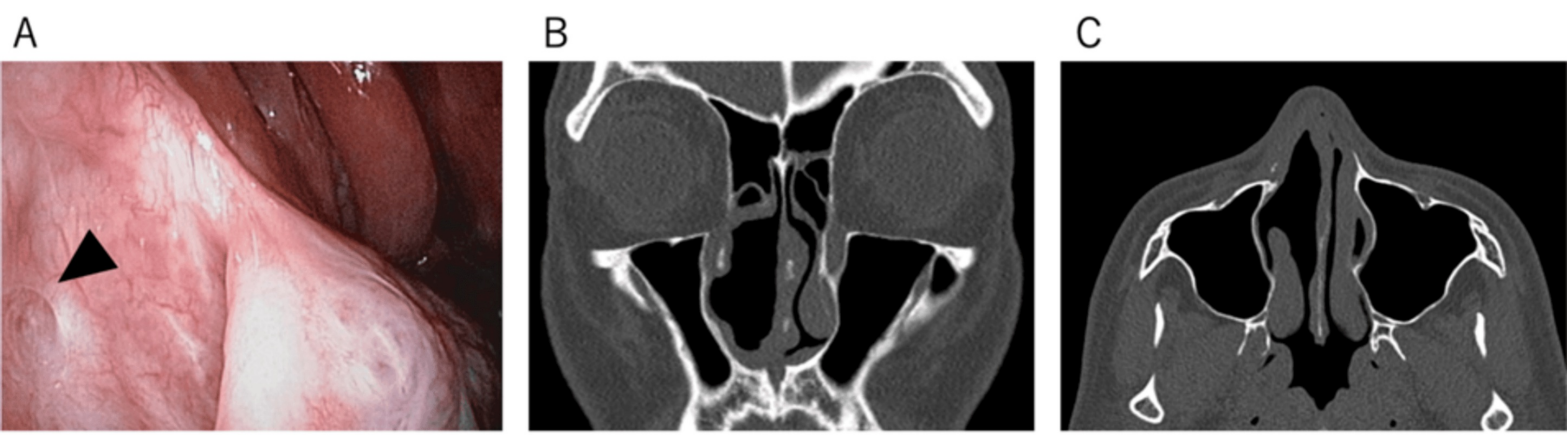
Findings at the 12-Month Postoperative Follow-Up (A) No evidence of tumor recurrence. The black arrowhead indicates the opening of the nasolacrimal duct. (B-C) CT images show no radiological findings suggestive of recurrence. CT, computed tomography

These findings confirmed the diagnosis of an SFT, and no adjuvant therapy was administered thereafter. The patient remained disease-free at 12 months of follow-up, with nasal endoscopy and CT confirming no evidence of recurrence or residual tumors (Figures 3A-3C).

## Discussion

Sinonasal SFTs represent a rare subset of head and neck SFTs, accounting for less than 2% of all reported cases [7]. Most patients present with unilateral nasal obstruction, swelling, or epistaxis [9]. Because of its indolent growth and nonspecific symptoms, sinonasal SFT is often misdiagnosed as a benign nasal polyp or vascular lesion.

Sinonasal SFTs have been described in approximately 50 reported cases, with most demonstrating favorable outcomes following surgical resection. However, within these published cases, only one has been explicitly documented as originating from the nasal vestibule [9]. Therefore, this report likely represents the second documented case worldwide, underscoring the extreme rarity of SFT at this anatomic location.

Histologically, SFT consists of spindle-shaped cells arranged in a disorganized manner, featuring varying levels of cellularity and staghorn-like branching vessels. The immunoprofile - CD34-positive, STAT6-positive, S-100/desmin-negative - is highly characteristic [6,10]. The discovery of the NAB2-STAT6 fusion gene by Robinson et al. [7] established a molecular foundation for the diagnosis of SFT. Demicco et al. proposed a risk stratification model that incorporated age, tumor size, and mitotic index. Based on these variables, patients were categorized into three groups: low-, intermediate-, and high-risk. No metastases were observed in the low-risk group, whereas the intermediate-risk group showed a 7% metastatic rate at 10 years, and the high-risk group demonstrated a 49% metastatic rate at five years [11]. In addition, Yamamoto et al. reported that, regardless of the primary site, mitosis, necrosis, and a Ki-67 index greater than 5% were significantly associated with recurrence, with Ki-67 being a particularly useful predictor [12].

Radiologically, sinonasal SFTs typically appear as well-circumscribed, enhancing masses without bony destruction, consistent with the findings reported by Yang et al. [13] and those of our case. In our patient, CT revealed a homogeneous soft-tissue mass that displaced the nasal septum toward the contralateral side and compressed adjacent structures, without evidence of bony erosion or infiltration. MRI revealed a mildly lobulated, well-demarcated lesion with iso- to slightly hyperintense signals on T1WI and heterogeneous hypointensity on T2WI. DWI revealed an isointense signal, and gadolinium-enhanced T1WI revealed homogeneous enhancement. Notably, internal linear flow voids and strongly enhanced vascular structures were observed, consistent with feeding vessels. On PET-CT, the lesion showed mild FDG uptake (SUVmax 2.2), supporting a low-to-intermediate metabolic profile in a hypervascular, well-circumscribed tumor.

Because hypervascular masses in the anterior nasal cavity/vestibule can mimic several entities, a focused differential diagnosis is crucial. Vascular lesions (e.g., hemangiomas or lobular capillary hemangiomas) can show avid enhancement but typically lack characteristic spindle-cell morphology and STAT6 nuclear positivity. Glomangiopericytoma is a key histologic mimic of sinonasal tract tumors; however, it generally does not exhibit diffuse nuclear STAT6 expression and more often shows smooth muscle marker expression (e.g., α-SMA). Schwannomas and other peripheral nerve sheath tumors usually show S-100/SOX10 positivity, whereas smooth muscle tumors demonstrate desmin and/or α-SMA positivity. Accordingly, integrating the radiologic impression of a well-demarcated, hypervascular lesion without bony destruction, with the characteristic immunoprofile - particularly diffuse nuclear STAT6 positivity and CD34 positivity, with S-100/desmin negativity - provides a strong discriminatory value for diagnosing SFT [6,7].

Regardless of the primary site, SFT is generally managed with curative surgical resection [14]. Although en bloc removal is ideal, carefully performed piecemeal endoscopic resection can yield complete local control, provided that the margins are clearly visualized and the tumor is completely resected. Our case corroborates these observations: despite the confined vestibular space, complete macroscopic excision was achieved, and no recurrence was observed at 12 months postoperatively.

For unresectable or metastatic disease, Akbulut et al. [15] demonstrated favorable responses to anti-angiogenic agents, such as temozolomide-bevacizumab and pazopanib, offering promising systemic options.

Given the potential for very late recurrence or metastasis, sometimes more than 10 years after the initial treatment, long-term follow-up is essential. We recommend periodic nasal endoscopy and systemic imaging (CT or chest radiography) for at least five years, even in low-risk cases such as this one.

## Conclusions

An SFT of the nasal cavity is rare, and its origin in the nasal vestibule is exceptionally uncommon, to our knowledge, with only one case documented in the literature. Diagnosis requires immunohistochemical confirmation, particularly of nuclear STAT6. Even when en bloc excision is not feasible, meticulous piecemeal endoscopic removal can achieve complete clearance and excellent local disease control. Although uncommon, distant metastasis has been reported in head and neck SFTs; therefore, long-term follow-up is indispensable.
